# Vis-NIR and SWIR hyperspectral imaging method to detect bruises in pomegranate fruit

**DOI:** 10.3389/fpls.2023.1151697

**Published:** 2023-04-21

**Authors:** Emmanuel Ekene Okere, Alemayehu Ambaw, Willem Jacobus Perold, Umezuruike Linus Opara

**Affiliations:** ^1^ SARChI Postharvest Technology Research Laboratory, Africa Institute for Postharvest Technology, Faculty of AgriSciences, Stellenbosch University, Stellenbosch, South Africa; ^2^ Department of Electrical and Electronic Engineering, Stellenbosch University, Stellenbosch, South Africa; ^3^ UNESCO International Centre for Biotechnology, Nsukka, Enugu, Nigeria

**Keywords:** pomegranate fruit, non-destructive testing, hyperspectral imaging, Vis-NIR, SWIR, bruise detection, pattern recognition

## Abstract

**Introduction:**

Fresh pomegranate fruit is susceptible to bruising, a common type of mechanical damage during harvest and at all stages of postharvest handling. Accurate and early detection of such damages in pomegranate fruit plays an important role in fruit grading. This study investigated the detection of bruises in fresh pomegranate fruit using hyperspectral imaging technique.

**Methods:**

A total of 90 sample of pomegranate fruit were divided into three groups of 30 samples, each representing purposefully induced pre-scanning bruise by dropping samples from 100 cm and 60 cm height on a metal surface. The control has no pre-scanning bruise (no drop). Two hyperspectral imaging setups were examined: visible and near infrared (400 to 1000 nm) and short wavelength infrared (1000 to 2500 nm). Region of interest (ROI) averaged reflectance spectra was implemented to reduce the image data. For all hypercubes a principal components analysis (PCA) based background removal were done prior to segmenting the region of interest (ROI) using the Evince® multi-variate analysis software 2.4.0. Then the average spectrum of the ROI of each sample was computed and transferred to the MATLAB 2022a (The MathWorks, Inc., Mass., USA) for classification. A two-layer feed-forward artificial neural network (ANN) is used for classification.

**Results and discussion:**

The accuracy of bruise severity classification ranged from 80 to 96.7%. When samples from both bruise severity (Bruise damage induced from a 100cm and 60 cm drop heights respectively) cases were merged, class recognition accuracy were 88.9% and 74.4% for the SWIR and Vis-NIR, respectively. This study implemented the method of selecting out informative bands and disregarding the redundant ones to decreases the data size and dimension. The study developed a more compact classification model by the data dimensionality reduction method. This study demonstrated the potential of using hyperspectral imaging technology in sensing and classification of bruise severity in pomegranate fruit. This work provides the foundation to build a compact and fast multispectral imaging-based device for practical farm and packhouse applications.

## 1 Introduction

Pomegranate (Punica granatum L) is undeniably one of the most ancient deciduous fruit in the world (Al-Said et al., 2009; Opara et al., 2009; Pareek et al., 2015). With its origin traceable to the Middle East, it has expanded and has now been grown across the world, even meeting commercial export in South Africa (Adetoro et al., 2020; Pienaar and Barends-Jones, 2021). Pomegranate fruit can be consumed as fresh arils or in its processed form such as juice, dried arils, jams, etc. In the past decades, the demand for pomegranate fruit has been increasing due to its nutritional and health benefits (Lansky and Newman, 2007; Al-Said et al., 2009; Fawole and Opara, 2013). It has been recounted to be highly effective for preventing inflammatory diseases and induces anti-proliferative and antimetastatic side effects in human (Pareek et al., 2015).

Bruise is the most common type of postharvest mechanical injury affecting pomegranate fruit (Opara et al., 2021a; Opara et al., 2021b). Bruise reduces fruit quality and causes considerable post-harvest losses and decreases the income (Opara and Pathare, 2014; Shafie et al., 2017; Hussein et al., 2019). Bruise usually results when the fruit is subjected to high impact and vibration (Opara and Pathare, 2014; Shafie et al., 2017; Opara et al., 2021a). Bruise damage normally manifest when the outer tissue of the fruit fails without rupturing due to excessive mechanical stress (Ahmadi et al., 2014; Hussein et al., 2019; Opara and Pathare, 2014). various studies showed that most bruises occurred during harvest and transportation to the packhouse and during handling in the packaging processing line. Studies have shown the detrimental effect of bruise on the physical and biochemical quality of pomegranate fruit (Shafie et al., 2015; Hussein et al., 2019). The economic losses in the fruit and vegetable industry due to bruise damage is substantial (Van Zeebroeck et al., 2007; Opara and Pathare, 2014). In the pomegranate industry, bruise damage reduces the market value considerably and causes a huge economic loss (Opara et al., 2021a; Opara et al., 2021b), as bruised fruits do not meet export quality and are devalued at marketplace.

Unlike other fruit with soft tissues and thin rind/peel such as apples and pear, early detection of bruises on pomegranate fruit is difficult due to the tough and leathery skin of this fruit (Hussein et al., 2019). Bruise on pomegranate fruit is only visible long after the impact (Hussein, 2019). Typically, in the industry, bruises are identified through visual inspection by trained panels or line operators and removed manually. This approach for bruise diagnosis is laborious, time consuming and subjective. Therefore, there is a need for alternative technology for rapid and non-destructive detection of early bruise damage. Other studies showed that pomegranate fruit responded physiologically and in some physico-chemical changes when they undergo bruises. This is indicative in the changes in total soluble solids (TSS), titratable acidity (TA), Brix-to-acid ratio (TSS : TA) and BrimA when exposed to bruising (Hussein, 2019). The effect of fruit ripeness (maturity), on bruise susceptibility has been reported (Hussein, 2019; Hussein et al., 2019), with corresponding physico-chemical changes. The ripening (maturity) stage, depending on the type of fruit and cultivar, can be the most important factors influencing bruise damage susceptibility (Hussein, 2019; Hussein et al., 2019). Previous studies have revealed that mature fruits are more susceptible to bruise damage than immature fruit (Xing and De Baerdemaeker, 2005). Spectroscopic analysis is gaining widespread research attention because of its ability to extract huge chemical information to analyze and develop a quality prediction model for several fruit types (Xing and De Baerdemaeker, 2005; Du et al., 2020).

There have been different imaging and feature extraction approaches for fruit bruise detection and measurement (Shahin et al., 2002; Kim et al., 2014; Du et al., 2020; Zeng et al., 2020). The shortcoming with most of these approaches is the need for wider spectral range (Xing and De Baerdemaeker, 2005). Spectroscopic assessment for fruit quality gained attention in research as viable nondestructive technique for quality attributes and grading (Khodabakhshian et al., 2017; Arendse et al., 2018; Jamshidi et al., 2019). Other imaging techniques that have been applied for bruise detection in recent times include X-ray (Hussein, 2019), Thermal imaging (TI) (Zeng et al., 2020), Magnetic resonance imaging (MRI) (Razavi et al., 2018), Fluorescence imaging (FI) (Chiu et al., 2015; Everard et al., 2016) as well as hyperspectral imaging (Dian et al., 2019; Zhu et al., 2016).

Hyperspectral imaging (HSI) has emerged as a powerful non-destructive inspection technique in the agricultural, biosecurity diagnostic and food domain recently. HSI is a non-invasive/nondestructive technique that integrates spectroscopy and imaging to form one system (Wu and Sun, 2013; Su & Sun, 2018). This non-destructive approach has been proposed for detections of different fruit defects (Arendse et al., 2021; Okere et al., 2021). It has been employed for disease detection (Li et al., 2016; Siedliska et al., 2018), common defects (Li et al., 2013; Zhang et al., 2015; Munera et al., 2021), physical damage (Lee et al., 2014), and in particular for bruise detection (Che et al., 2018; Tan et al., 2018b; Fang et al., 2019; Zhu & Li, 2019). Some of the specific fruits that have been investigated for bruise damage includes apples (Siedliska et al., 2014; Ferrari et al., 2015; Li et al., 2018), strawberries (Nagata et al., 2006; Liu et al., 2018), blueberries (Jiang et al., 2016; Fan et al., 2018), peaches (Li et al., 2018), kiwifruit (Lü and Tang, 2012), pears (Dang et al., 2012; Fu and Wang, 2022), jujube (Feng et al., 2019), cucumbers (Ariana et al., 2006), and so forth. These studies reported success in accurate classification of bruise severity suggesting the potential of implementing the technique. However, to the best of our knowledge, no study has yet reported on the application of hyperspectral imaging for non-destructive detection and classification of bruise of pomegranate fruit. Therefore, this study seeks to explore the potential of hyperspectral imaging to detect and classify bruise severity for pomegranate fruit.

## 2 Materials and methods

### 2.1 Fruit procurement and sample preparation

In this study, pomegranate fruit (cv. Wonderful) was procured from Sonlia pack-house in the Western Cape region, South Africa. Sample pomegranates were harvested at commercial maturity at average maturity indices, viz. total soluble solids (TSS) of 16.36± 1.05°Brix and brix-acid ratio (TSS/TA) of 10.08± 2.13%. A total of 90 pomegranate fruit, with an average weight of 280 ± 45g, without visible surface defects were individually sorted, washed, and stored at 7.0 ± 1°C and 90 ± 2% RH, which is the recommended optimum storage condition for pomegranate fruit (Arendse et al., 2018).

### 2.2 Bruise simulation

**Figure 1:**
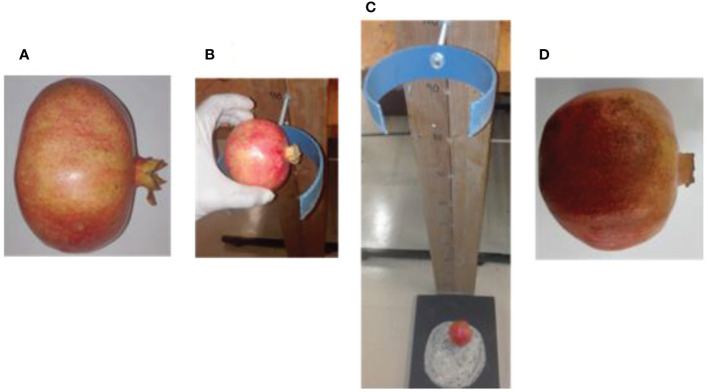
Picture of pomegranate fruit sample under drop impact bruise from 100cm height **(A)** fresh unbruised fruit sample **(B)** fruit placed at 100cm drop height **(C)** fruit dropped under free fall due to gravity **(D)** bruised fruit sample.

Bruise damage was created on the middle (equatorial) region of the fruit by dropping fruit from a predefined height onto a steel surface with side of the fruit perpendicular to the metal surface. This experiment follows the previously developed method by Hussein et al. (2019) (**Figure 1**). Each pomegranate fruit was dropped once from a given height to the metal surface and caught by hand after the first rebound to avoid multiple impacts. Following impact tests, fruit were incubated at ambient condition (19 – 22°C, 60 ± 5% RH) for an hour prior to image acquisition. A total of 90 pomegranates were used for this study. Samples were sub-divided into three groups of 30 samples, each representing dropping induced bruising level: 100 cm, 60 cm, and no drop (not bruised). Assuming the fall was nearly free, impact energies applied on the fruit surface were calculated according to impact force from falling object. The calculated average impact energy was approximately 760 ± 0.5 mJ and 680 ± 0.8 mJ for the falling from 100 cm and 60 cm heights, respectively.

### 2.3 Hyperspectral image acquisition system

**Figure 2:**
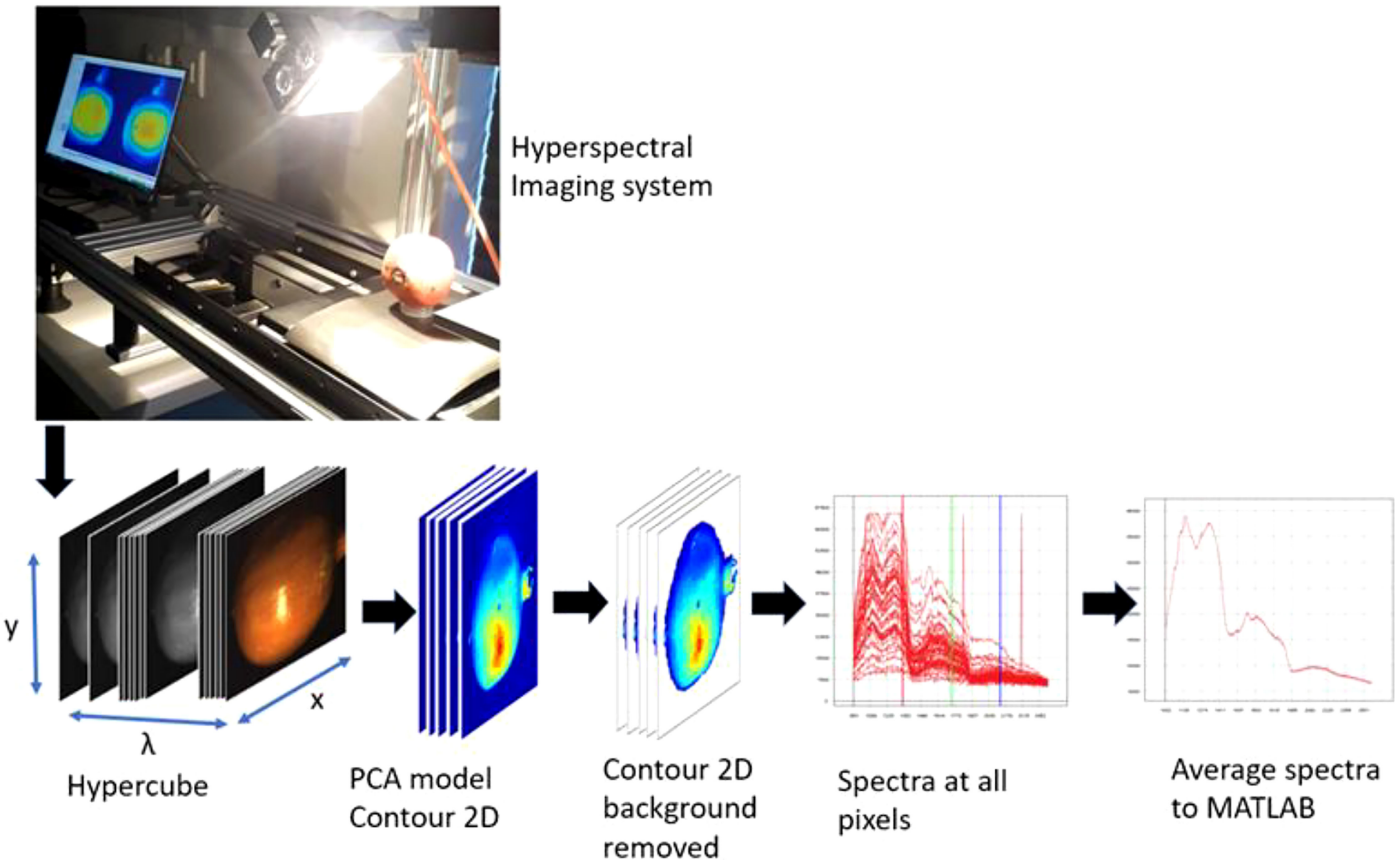
Schematics illustrating the hyperspectral imaging and analysis workflow followed in this study.

Prior to image acquisition the system was set up as follows. The distance between sample and camera was set to 20.5 cm; the grey standard was fixed at 68 mm from above Scanning was performed at the Central Analytical Facility (CAF) Vibrational Spectroscopy Unit of Stellenbosch University. Two different hyperspectral imaging cameras: HySpex VNIR-1800 and HySpex SWIR-384 (NEO; Norsk Electro Optikk, Norway) were tested (**Figure 2**). The camera specifications for both equipment is elaborated and compared in (**Table 1**). In the VNIR camera, images are acquired at wavelengths ranging from 400 to 1000 nm with a waveband of 186 and spectral resolution of 3.26 nm. **Figure 2** illustrates the hyperspectral image acquisition system and the formation of three‐dimensional hyperspectral data (hypercube). The VNIR has spatial pixels (x) of 1800 which corresponds to the number of photodetectors along the spatial dimension of the detector array of the camera. The second spatial dimension (y) is the number of pixels in the scanning direction and is physically bounded by the size of the scene and the speed of the translation stage. A 30 cm focal length lens with field view of 9.733 cm were used. Reflectivity reference data were obtained for each fruit. Hence, each image was obtained as a three-dimensional image block (x, y, λ), including 1800 × y pixels on the space dimension (x, y), and 128 bands at 3.26‐nm intervals within a range of 400 to 1000 nm on the spectral dimension (λ).

**Table 1:** Summary of hyperspectral imaging system, comparison of SWIR and VNIR camera specifications.

Main specifications	SWIR	VNIR
Spectral range	930 – 2500 nm	400 – 1000 nm
Spatial pixels	384	1800
Spectral channels	288	186
Spectral sampling	5.45 nm	3.26 nm
FOV	16°	17°
Pixel FOV across/along	0.73/0.73 mrad	0.16/0.32 mrad
Bit resolution	16 bit	16 bit
Noise floor	150 e-	2.4 e-
Dynamic range	7500	20000
Peak SNR (at full resolution)	>1100	>255
Max speed (at full resolution)	400 fps	260 fps
Power consumption	30 W	30 W
Dimensions (l-w-h)	38 – 12 – 17.5 cm	39 – 9.9 – 15 cm
Weight	5.7 kg	5.0 kg
Camera interface	Camera Link	Camera Link

The SWIR camera works at a wavelength range of 950 to 2500 nm with spectral wavebands of 288 and spectral resolution of 5.45 nm. It has spatial pixels of 384. The cameras were mounted above a translation stage which has a speed regulation system (**Figure 2**). A 30 cm focal length lens with a field view of 9.470 cm was used. Reflectivity reference data were obtained for each fruit with the bruised surface facing the camera.

### 2.4 Hyperspectral image calibration

To minimize the impact of the uneven intensity distribution of the light source and dark current in the charge coupled device (CCD) detector on the hyperspectral images, image correction was performed using known true spectral information. Eqn. (1) provides the formula for the image correction.

ρ_


(1) $${\text{ρ}}_{\text{xy}}\left(\text{λ}\right)={\text{ρ}}_{\text{ref}}\left(\text{λ}\right)\frac{{\text{R}}_{\text{xy}}\left(\text{λ}\right)-{\text{R}}_{\text{dark}}\left(\text{λ}\right)}{{\text{R}}_{\text{ref}}\left(\text{λ}\right)-{\text{R}}_{\text{dark}}\left(\text{λ}\right)}$$

where *ρ_ref_(λ)* is the reflectivity of the 50% grey calibration plate (Zenith Polymer^®^ Reflectance standard; SphereOptics GmbH, Germany); *R_xy_(λ)* is the original uncorrected hyperspectral image; *R_ref_(λ)* the image of the calibration board and *R_dark_(λ)* is the completed black image collected after turning off the light source and *ρ_xy_(λ)* the spectra of the corrected image. The system operation and image acquisition were carried out using ‘Breeze’ software (version 2021.1.5, Umeå, Prediktera, Sweden) installed on a 64-bit Dell computer of 40 GB RAM and processor speed of 2.20GHz running on Windows 10 pro–operating system.

### 2.5 Explorative analysis using PCA

The three-dimensional hyperspectral images (hypercubes) were imported into Evince software (version 2.7.10, Prediktera, Sweden) for pre-processing and background removal. The background was removed by interactively separating (selecting, excluding, and reconstructing) the background pixels from the fruit pixels using contour 2D and scatter 2D plots of the PCA applied on individual and group hypercubes (**Figure 2**).

Preprocessing of extracted hyperspectral image data is necessary to reduce artifacts (variations that are not required in the spectral data) arising due to background noise, instrumental effects or luminescence and heterogeneity in samples (shape, size and position of sample) (Magwaza et al., 2012; Xu et al., 2023). The hypothesis is that the part of the spectral signal removed represents an interference and is generally not useful for numerical analysis. Different spectral preprocessing algorithms have been employed individually or in a sequential processing mode to reduce artifacts (Magwaza et al., 2012; Ravikanth et al., 2017). In this study raw reflectance data and six commonly used spectral preprocessing, namely, multiplicative scatter correction (MSC), standard normal variate (SNV), de-trending (DT), continuum removal (CR) and Savitzky–Golay first and second derivative were compared to identify the best for predicting bruise severity level. The SNV model achieved the best classification predictive performance compared to other methods used. SNV reduces disturbances in spectral data by correcting spectra with the mean and standard deviation of each spectrum (Tan et al., 2018a).

Subsequent hyperspectral data processing was implemented using hyperspectral Imaging Library in MATLAB^®^ (The MathWorks, Inc., Natick, Massachusetts, United States). Supervised classification models based on a two-layer feed-forward artificial neural network (ANN), with sigmoid hidden and softmax output neurons was used to classify inputs into two target (for bruise detection) and three target (bruise severity) categories (Jamshidi, 2003; Nturambirwe and Opara, 2020). The original data was randomly divided into training set (70%), validation (15%) and test set (15%). Training set is presented to the network during training, and the network is adjusted according to its error. Validation set measure network generalization, and halt training when generalization stops improving. Testing set has no effect on training and so provide an independent measure of network performance during and after training. To achieve this, a dummy binary-coded matrix of equal rows as the input was created. In this study, for the case of bruise detection, 2-column response matrix in which samples belonging to the first class (bruised) were described by a vector [1 0] while the No bruise class was represented by the vector [0 1]. In the case of severity, a 3-column matrix was generated with the first class (60 cm drop) described by [1 0 0], 100 cm drop [0 1 0], and the No drop class [0 0 1] respectively. Classification was accomplished by using the machine learning and deep learning functions in MATLAB. Classification performances were evaluated based on its overall classification accuracy for training set, test set and validation set as well as class error. A good model should possess high classification accuracy and low-class error. A model with a 100% classification accuracy means that the model made no classification error.

## 3 Results and discussion

### 3.1 Principal component analysis

**Figure 3:**
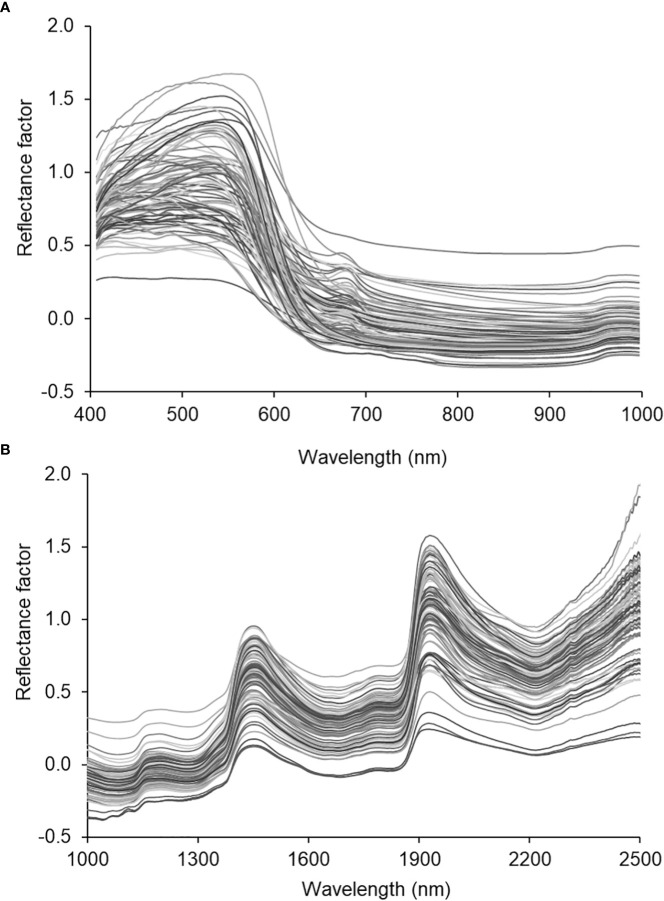
Spectral characteristic curves of the SWIRL data. average spectra of the hyperspectral images of all samples.

**Figure 3** depicts the averaged spectral of all the samples scanned with the VNIR (**Figure 3A**) and SWIR (**Figure 3B**) cameras squeezed out using the Evince software (version 2.7.10, Prediktera, Sweden). Evince extracted the spatial (horizontal and vertical), and spectral profiles from the image display. Each sample fruit exhibited a unique spectral signature based on the sample’s composition, surface structure, viewing geometry, etc. The assumption is that bruising can create its own signature by affecting the surface structure and composition. However, the overall shape (locations of wavelength bands where highs and lows) is similar across the electromagnetic spectrum for all samples in both cameras. Hence, the classification parameter this study used to identify bruise severity and presence/absence of a bruise was based on reflectance values at bands than the overall shape of the spectra.

**Figure 4:**
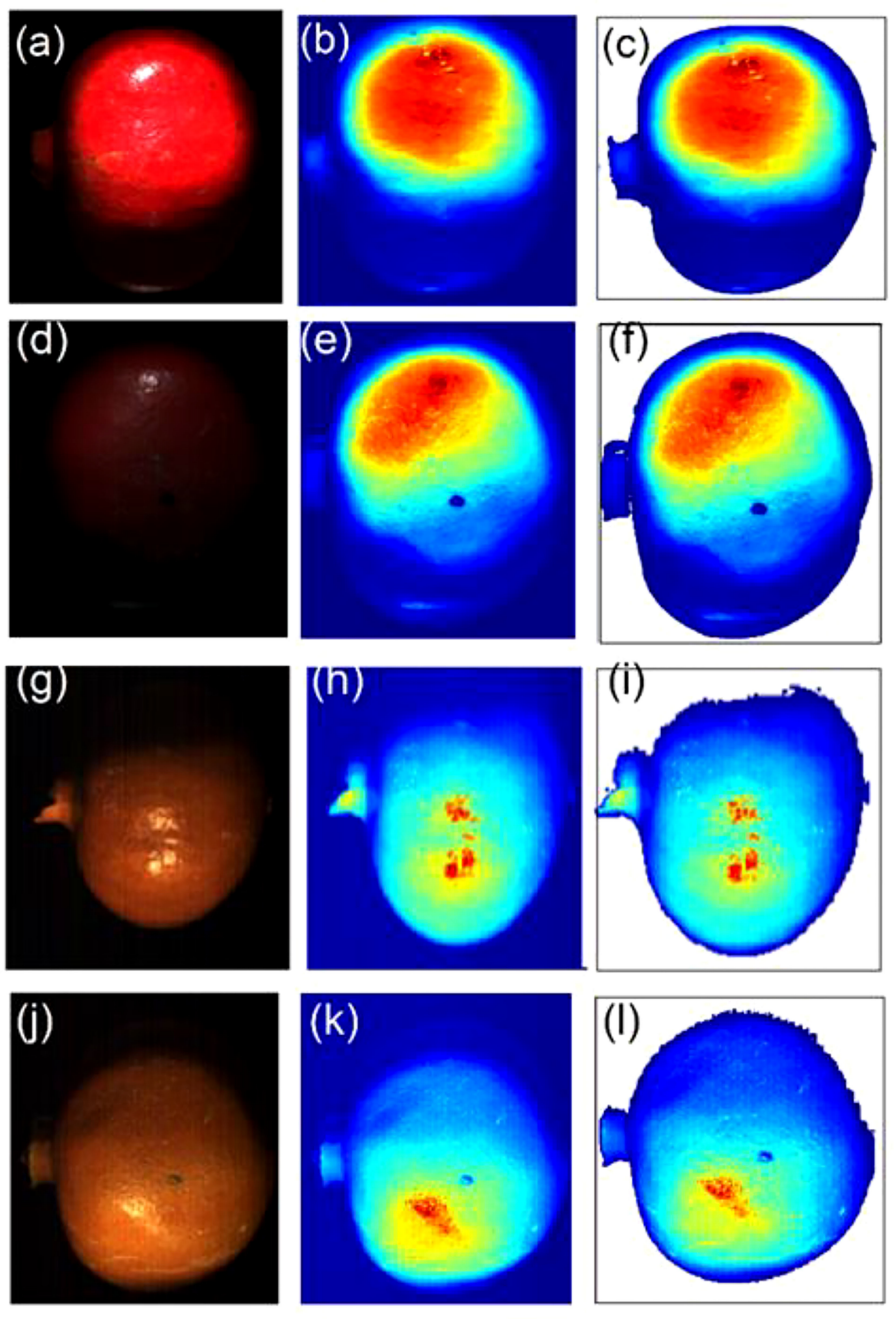
A typical explorative PCA analysis. Sample with no drop **(A–C)** and drop from 100 cm **(D–F)** under the VNIR camera and no drop **(G–I)** and drop from 100 cm **(J–L)** under the SWIR camera.

The two cameras have their distinctive spectra depending on their spatial and spectral resolution (**Table 1**). Due to its high spatial resolution the VNIR camera provided high-resolution HS images as shown by (**Figures 4A–F**) for sample without bruise and with bruise, respectively. The SWIR camera, due to its law spatial resolution, provides rough images with noticeable spatial lines on the painting (**Figures 4G–I**) and (J-L)). Correspondingly, the HS image visualization and data analysis process is much faster and easier for SWIR than VNIR. Using the Evince software the initial data compression stage was undertaken by cropping the view span to capture fruit only, PCA based background removal. This process compressed the data size significantly. In average, the HSI data size reduced from 3.5 GB to 200 MB for the VNIR and 1.5 GB to 150 MB for the SWIR cameras, respectively, before transferring to MATLAB.

**Figure 5:**
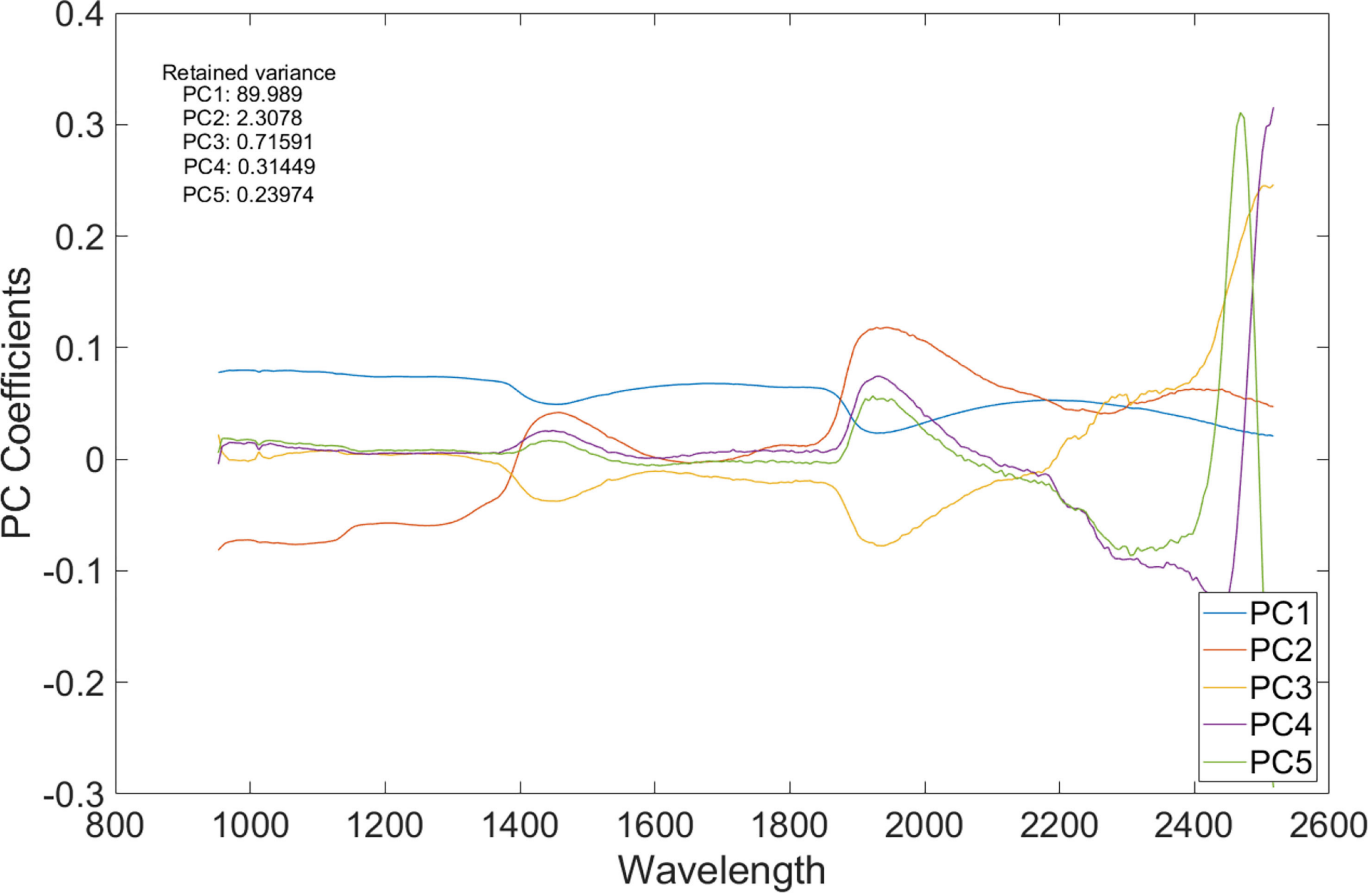
A plot of principal component analysis (PCA) coefficients *vs.* wavelength of the SWIR HS image.

For each sample, the number of spectrally distinct endmembers were estimated using the find the number of endmembers present in a hyperspectral data cube feature by using the noise-whitened Harsanyi–Farrand–Chang (NWHFC) method implemented in MATLAB, and the corresponding bands were identified using PCA method for dimensionality reduction (**Figure 5**). Effective band selection was done for each fruit sample.

**Figure 6:**
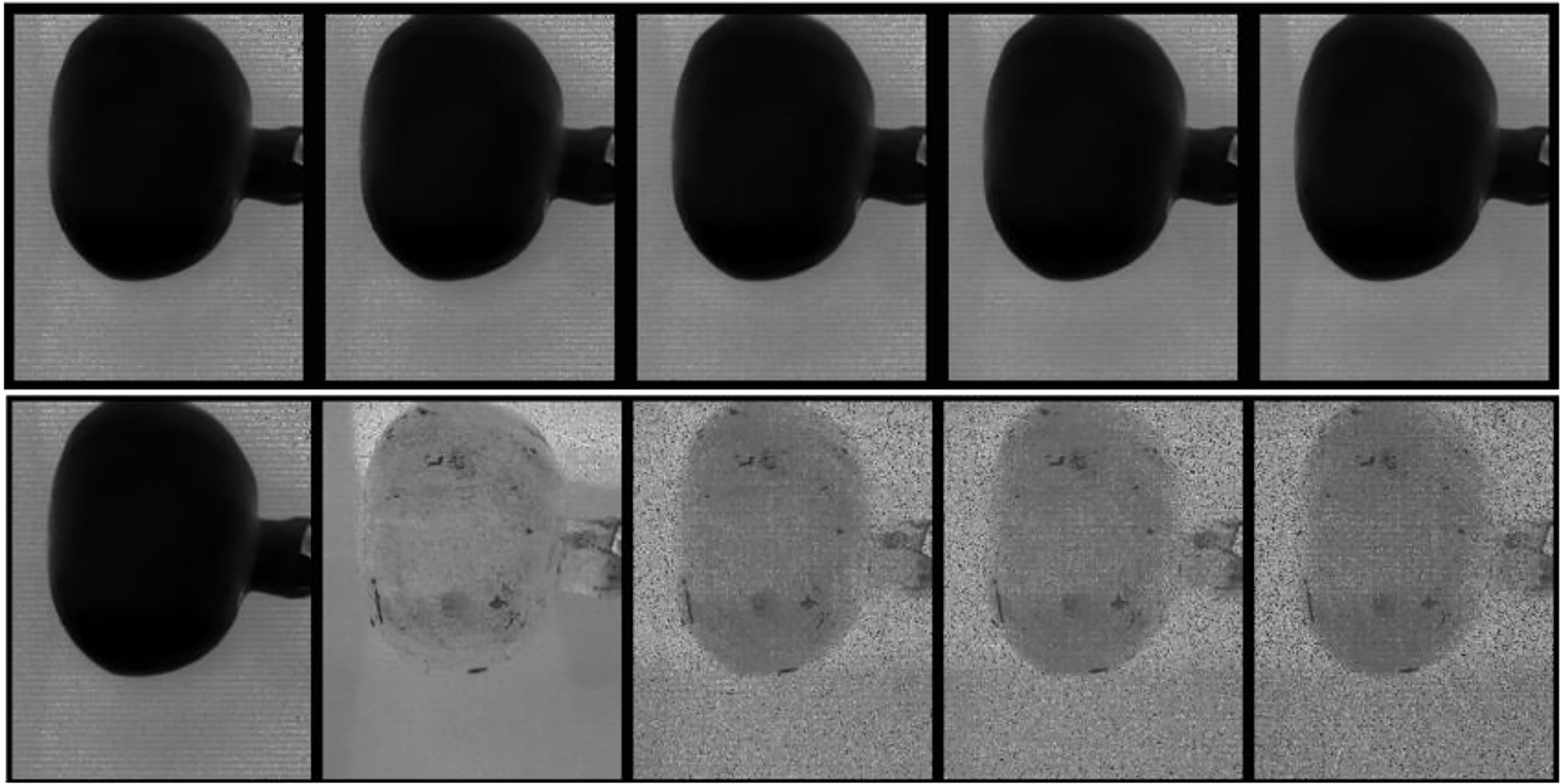
Display of the first 5 spectral bands in the input data cube (top row) and the five most informative bands (bottom row) of a typical fruit without bruise.

**Figure 7:**
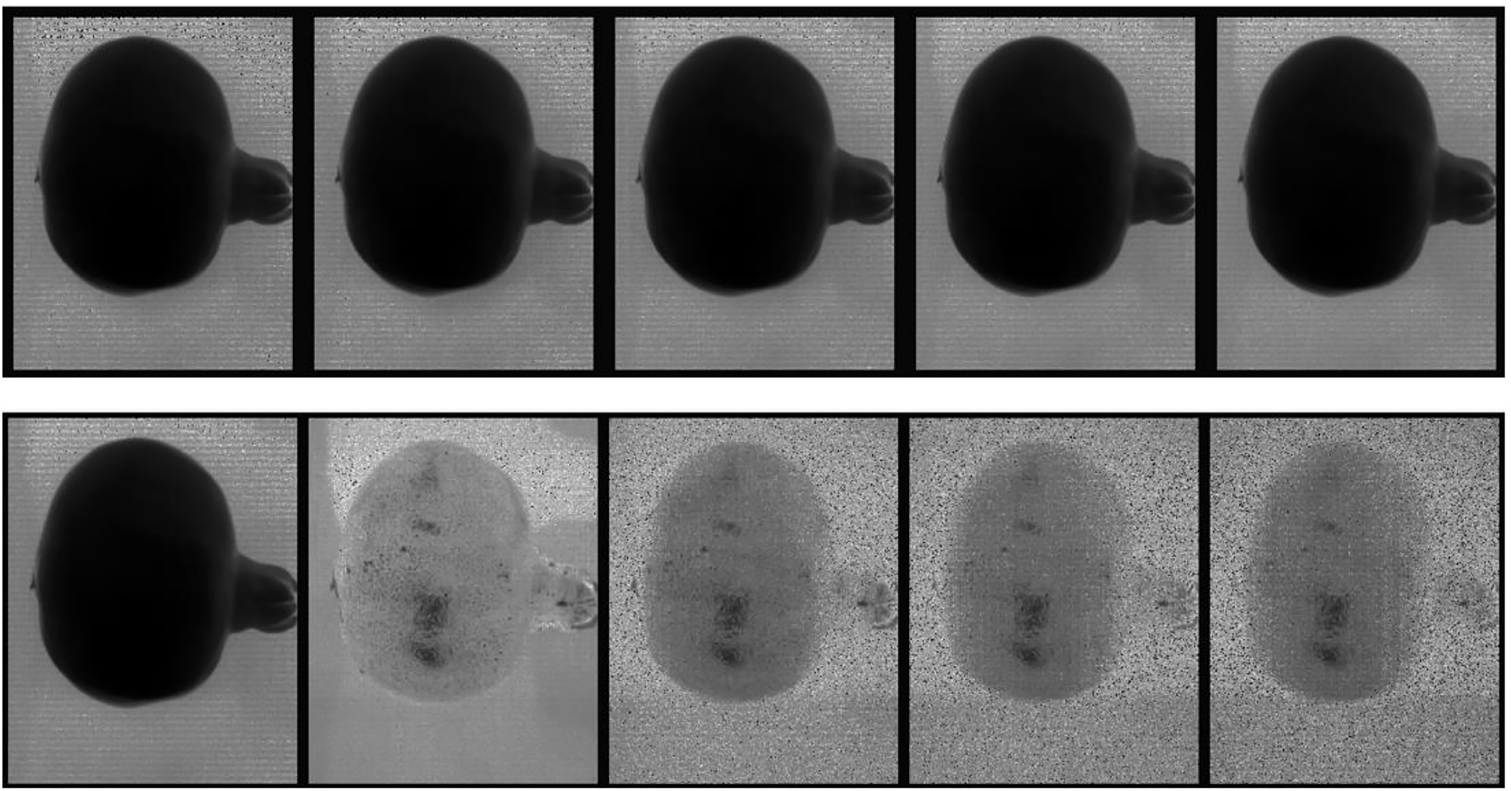
Display of the first 5 spectral bands in the input data cube (top row) and the five most informative bands (bottom row) of a typical fruit sample bruised from falling from 100 cm.

**Figure 8:**
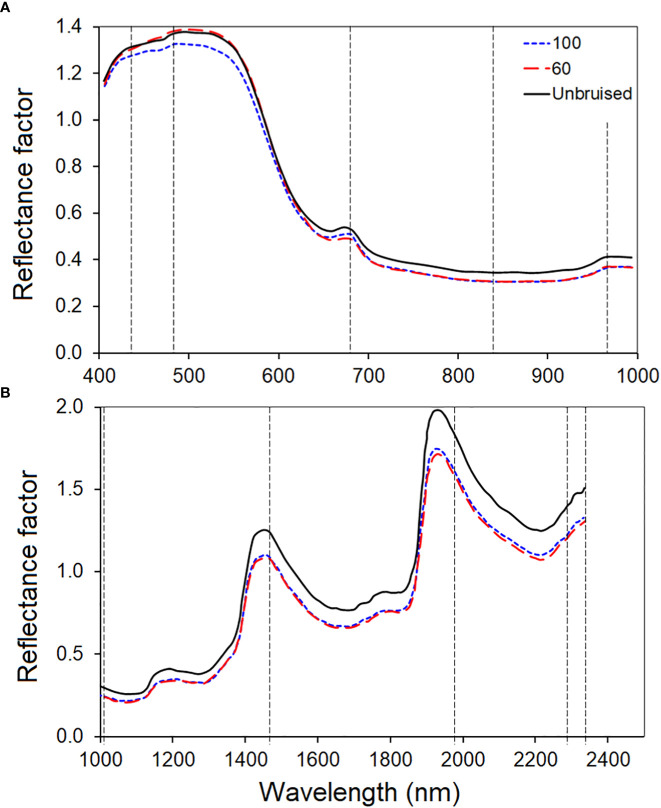
The average class spectral of the three bruise severity classes of the VNIR **(A)** and SWIR **(B)** camera. The vertical dashed lines identified the most informative bands selected by the effective wavelength (407, 639 and 917nm) selected using the noise-whitened Harsanyi–Farrand–Chang (NWHFC) method.

**Figure 6**, top row, displays the first five spectral bands of the original data of unbruised fruit. Variability is not significant both between bands and spatially on the fruit surface. **Figure 6**, bottom row, shows the same fruit seen with the identified five informative bands. Clearly, differentiations come both spatially and spectrally with the informative bands. The same informative bands used on a fruit that was bruised by dropping from 100 cm height is shown in **Figure 7**. The accentuation of the bruise mark in the bottom raw (viewed with the informative bands) is apparent. The residences of the five effective bands are shown as vertical dashed line on the class mean spectra of the two cameras (**Figure 8**).

The class mean spectral plot clearly distinguished the two bruised groups from the unbruised group. The unbruised spectral (orange) showed the highest reflectance signature across the electromagnetic spectrum of the SWIR camera, while both bruised samples at different severities (blue and yellow) showed lower reflectance. However, for the VNIR camera, the variation between classes looks small and it is not consistent across the spectrum. Similar trend is observed in most bruise study for different fruit (Siedliska et al., 2018; Tan et al., 2018b). This spectral profile pattern for bruised and unbruised samples has been attributed to the fact that there is an outflow of water from the surface of the sample that have been bruised (Siedliska et al., 2018; Tan et al., 2018b).

### 3.2 Classification model development for bruise fruit detection

The classification test results for bruise detection classification accuracy, true positive, false positive of the VNIR and SWIR data are summarized on **Table 2**. The results for classification were grouped into three groups or levels of severity, group 1 comprised fruit bruised at 60 cm and unbruised fruit, group 2 was made up of samples bruised at 100 cm and unbruised samples and finally group 3 which combined the two-bruised fruit samples (60 cm *vs.* 100cm). For bruise severity classification training, each ROI averaged reflectance values at the five wavelengths (1 × 5), presented to the classification model, is accompanied by a (1 × 3) target matrix where each column indicates a category with a one in either element 1, 2, or 3, defining the desired network output (no bruising, bruised at 60 cm and 100 cm). On the other hand, the bruise classification problem, which is a binary (two-class) problem distinguishing between bruised and unbruised samples, is accompanied by a (1 × 2) target matrix where each column indicates a category with a one in either element 1 or 2. The ANN pattern recognition algorithm divides the data randomly into training (70%), testing (15%) and validation (15%) sets during model development.

**Table 2:** Summary of results for the different bruise severity of pomegranate fruit.

Type	Spectra range	Test set			
		Sample number	Correct class	Incorrect class	Accuracy (%)
Sound (Unbruised)	SWIR VNIR	30 30	27 29	3 1	90 96.7
Bruised at 60cm	SWIR VNIR	30 30	23 25	7 5	76.7 83.3
Bruised at 100cm	SWIR VNIR	30 30	29 27	1 3	96.7 90
Combined 60cm and 100cm	SWIR VNIR	30 30	22 27	8 3	73.3 90

The effect of the structure of the artificial neural network (number of hidden neurons and random division of sample into training, testing and validation sets) on the performance of the classification was evaluated using error histogram, confusion matrix and Receiver Operating Characteristic curve. Confusion matrix is a very popular measure used while solving classification problems and it is used in this paper to report the classification performances. For the bruise severity classification which has three classes, the confusion matrix is a 3 x 3 and the bruise classification, which is binary, has a 2 x 2 confusion matrix.

#### 3.2.1 Classification performance for SWIR camera

**Figure 9:**
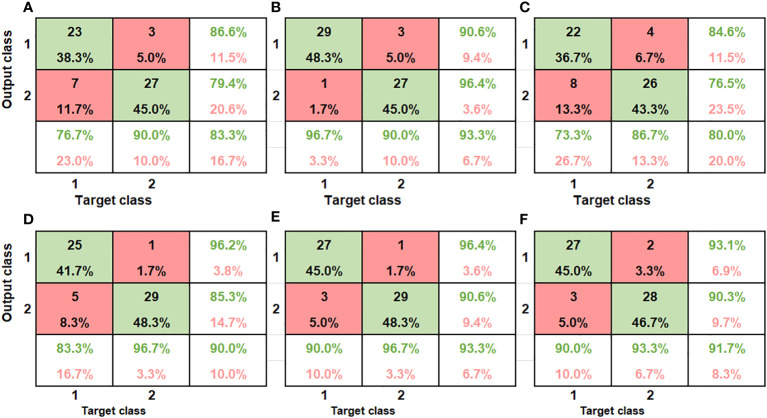
The confusion matrix of the classification performance of the different class groups using the SWIR camera (SI **(A)**, SII **(B)**, and SIII **(C)**, and VNIR SI **(D)**, SII **(E)** and SIII **(F)**. The x axis refers to the true categories, and the y axis refers to the classifier outputs. The integers in the matrix show several samples. The color encodes the percentage of a class of blocks (x) classified into a predicted class (y).

The ANN model accurately discriminated between bruised fruit from this group against unbruised ones (**Table 2**). The confusion matrix indicates how the model correctly and wrongly placed input data to the different severities is seen (**Figures 9A–C**). For the first severity stage SI (60cm drop height), the model showed a recognition accuracy of bruised samples and unbruised samples to be 76.7% and 90% respectively. The last column of the matrix indicates the ratio of the number of correctly classified samples to the number of all the total samples classified (**Figure 9A**). In the first column, for a total of 30 bruised samples, 23 were correctly classified as bruised while 7 were misclassified as unbruised. In the second column, out of the 30 unbruised samples, 27 were correctly recognized as unbruised while only 3 samples were misclassified. This yielded an overall recognition accuracy of 83.3% and a classification error of 16.7%.

Similar accuracy was obtained by Zhang and Li (2018). The authors implemented the Adaboost algorithm to investigate bruises on apple. The accuracy of their training model was 80.56%. The performance of the second severity group is presented (**Figure 9B**). The classification accuracy for this severity level (SII) improved as compared to the severity level I (SI). The average recognition accuracy improved from 83.3% (**Figure 9A**) to 93.3% (**Figure 9B**). The same accuracy was maintained for the unbruised samples, but a higher accuracy was obtained as 29 of the samples bruised under 100cm drop height were rightly classified. For the third category, SIII, comprising of samples bruised at 60cm height (30) and those bruised at 100cm height (30) from both SI and SII respectively were combined, model showed an average classification accuracy of 80% (**Figure 9C**). model performance showed high false positive and true negative of 8 out of 30 samples for 60cm drop bruised samples and 4 out of 30 samples for 100cm drop bruised samples. This shows model accurately classified SII (86.7%) data as compared to SI data (73.3%).

#### 3.2.2 Classification performance for VNIR camera

The results for the model recognition accuracy are listed in **Table 2**. Different model accuracy for the two different severity levels is shown (**Figures 9D–F**). As can be seen from the results, for bruise severity category one (SI), the VNIR model slightly outperformed the SWIR model, achieving an accuracy of 83.3% and 96.7% for bruised and unbruised samples (**Figure 9D**). The confusion matrix shows that for 30 samples bruised from a drop height of 60cm, 25 were rightly recognized while 5 were wrongly classified. The second column indicates that only 1 of the 30 unbruised samples was wrongly classified. This resulted in an average classification of 90% and class error of 10%.

For the case of severity category two (SII), the model showed similar performance to the SWIR, achieving an equal average accuracy of 93.3%. Unlike the SWIR, the model mis-classified 3 samples of 100cm dropped bruise samples out a total of 30 samples and correctly classified 27, achieving a 90% accuracy and class error of 10% (**Figure 9E**). The study on kiwifruit when applying VNIR-HSI system for bruise detection resulted similar low classification error of 14.5% (Lü & Tang, 2012). For the VNIR camera, it can be observed that the unbruised samples were always better recognized compared to the bruised data, irrespective of the bruise severity.

Similar trend was observed in several studies on bruising and detection of other defects on pome fruits (Zhang et al., 2015; Che et al., 2018). The result indicate that model was able to achieve higher accuracies as the severity heightened, this was contrary to findings by (Tan et al., 2018a). The authors re-ported lower identification accuracy for severely bruised samples. Both cameras performed equally as they both obtained an average accuracy of 93.3%.

The confusion matrix for model performance for a combined data is presented in (**Figure 9F**). model showed higher recognition accuracy for SII samples (93.3%) as compared to SI (90%). The VNIR data set performed slightly better than the SWIR when both bruised samples were grouped together. The average classification accuracy for the VNIR was 91.7% while that of the SWIR was 80%. The result indicates that the model was able to recognize the different bruise severity when they are modelled against each other. Some of the reasons for model misclassification might be because of light scattering effect during image data acquisition (Tan et al., 2018a). The shiny nature of pomegranate fruit could have an impact of how light penetrates the fruit during imaging.

#### 3.2.3 Classification model development for combined data for bruise detection

**Figure 10:**
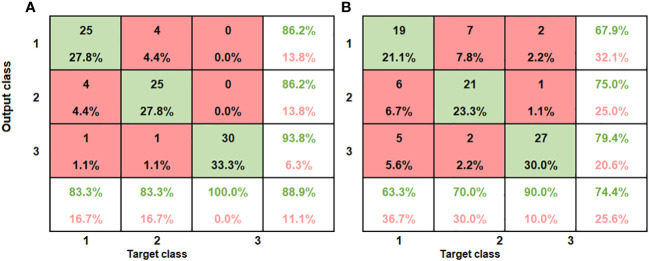
Summary of confusion matrices obtained for the combined ANN model for both SWIR and VNIR input data **(A)** SWIR classification performance **(B)** VNIR classification performance.

**Table 3** gives the combined classification performance of the ANN model for bruise detection of pomegranate fruit. **Figure 10** provides the resulting confusion matrix of the classification based on the ANN model. The columns of the matrix refer to the true categories, and the rows refer to the classifier outputs. For instance, for the SWIR (**Figure 10A**), of the 30 sample fruits in the first block (60 cm drop), 25 were correctly classified as “60 cm drop” 2 were classified as “100 cm drop” and 1 was classified as “No drop”. Of the 30 “100 cm drop”, 4 were wrongly classified as “60 cm drop,” 25 were correctly classified, and 1 was wrongly classified as “No drop.” Of the 30 “No drop”, all the 30 were correctly classified.

**Table 3:** Combined performance of the classification model for bruise severity detection on pomegranate fruit.

Drop distance (cm)	Combined model classification performance			
	Wavelength	Sample number	Accuracy (%)	Class error (%)
SI	SWIR VNIR	30 30	83.3 90	16.7 10
SII	SWIR VNIR	30 30	93.3 93.3	6.7 6.7
SI and SII	SWIR VNIR	30 30	80 91.7	20 8.3

The bottom row and the extreme-right column of the confusion matrix summarizes the performance of the classification model. Accordingly, the overall accuracy of the ANN model in classifying the SWIR data was 88.9% (classification error of 11.1%) and for the VNIR data the classification accuracy was 74.4% (classification error of 25.6%) (**Figure 10B**). This result agrees with the study on blackspot by (López-Maestresalas et al., 2016), where they concluded that SWIR achieved better results than VNIR data (98.56% against 95.46%). The results of sound samples classified as sound (true positives) (90% and 100%), were better than results for bruised samples classified as bruised (83.3%, 70%). This is the case for most reported study. Xing et al. (2005) reported 93% for non-bruised apples correctly classified and 86% accuracy for bruised samples.

Applying Adaboost algorithm for visual detection of bruises in apple (Zhang & Li (2018), observed out of the 54 samples of intact apples, 52 was correctly classified and only 2 was wrongly classified yielding an accuracy of 96.3%, while for the bruised samples, 87.04% was achieved. For jujube bruise detection (Feng et al., 2019), achieved almost 100% accuracy for healthy sample detection, in the NIR region, the authors attributed the lower accuracy for bruised samples to (browning coloration) of the bruised jujube samples which is like the healthy ones and made classification difficult.

Classification accuracies can also be impacted by the state of the sample, at the time of image acquisition. Huang et al. (2015), compared static and online application of multispectral data. The authors found classification accuracies to be higher for the static data (91.5%) as compared to the online (samples in motion on a translation stage) (87.3%).

## 4 Conclusion

This study investigates the detection and classification of bruises on pomegranate fruit surface using hyperspectral imaging system. The use of VNIR and SWIR cameras was explored. The result of the classification accuracy metric indicated that both cameras were able to accurately recognize bruised and unbruised pomegranate fruit samples. Both SWIR and VNIR data yielded highly accurate classification results ranging from 80% - 96.7%. The overall average classification accuracy achieved was 93.3% in distinguishing fruits dropped at 100cm and 90% for fruit dropped at 60cm height for the VNIR camera. Model performance was slightly lowered when both severity cases were combined, and model was able to accomplish a recognition accuracy of 80% and 91.7% for both SWIR and VNIR camera respectively. The model accuracy increases with the increase in bruise severity (93.3%). This study laid a foundation for further development of an in-line inspection system using hyperspectral imaging techniques for bruise detection on pomegranate fruits.

While gathering satisfactory datasets is very important, HS imaging tasks are still costly and time-consuming. Usually, HS image data sets are not enough for training artificial neural networks for classification model development. Using the raw HS image, as is, can easily create high dimensional data that can significantly cause overfitting. To augment this bottle neck, it is important to undertake data dimensionality reduction. This study implemented the method of selecting out informative bands and disregarding the redundant ones to decrease the data size and dimension. Unlike other fruit with soft tissues and surfaces, early detection of bruises on pomegranate fruit is difficult due to the tough and thick rind. Hence, developing an effective non-destructive technic like hyperspectral imaging could have a huge economic benefit in the industry. To this end, this paper demonstrated effective wavelength selection technique for a more compact and accurate classification prediction model. The implemented wavelength optimization technic will help develop a compact and fast multispectral imaging device for practical farm and packhouse applications.

## Data availability statement

The original contributions presented in the study are included in the article/supplementary material. Further inquiries can be directed to the corresponding author.

## Author contributions

Conceptualization, methodology, funding acquisition, project administration, supervision, and review and editing: UO; project administration and supervision: WP; investigation, validation, formal analysis, supervision, and review and editing: AT; data curation and writing—original draft preparation: EO. All authors contributed to the article and approved the submitted version.
